# Feedforward inhibition is randomly wired from individual granule cells onto CA3 pyramidal cells

**DOI:** 10.1002/hipo.22763

**Published:** 2017-07-24

**Authors:** Máté Neubrandt, Viktor János Oláh, János Brunner, János Szabadics

**Affiliations:** ^1^ Institute of Experimental Medicine Hungarian Academy of Sciences Budapest Hungary

**Keywords:** dentate gyrus ‐ CA3 interface, dentate gyrus, disynaptic inhibition, GABAergic inhibition, mossy fiber terminals, stratum lucidum

## Abstract

Feedforward inhibition (FFI) between the dentate gyrus (DG) and CA3 sparsifies and shapes memory‐ and spatial navigation‐related activities. However, our understanding of this prototypical FFI circuit lacks essential details, as the wiring of FFI is not yet mapped between individual DG granule cells (GCs) and CA3 pyramidal cells (PCs). Importantly, theoretically opposite network contributions are possible depending on whether the directly excited PCs are differently inhibited than the non‐excited PCs. Therefore, to better understand FFI wiring schemes, we compared the prevalence of disynaptic inhibitory postsynaptic events (diIPSCs) between pairs of individually recorded GC axons or somas and PCs, some of which were connected by monosynaptic excitation, while others were not. If FFI wiring is specific, diIPSCs are expected only in connected PCs; whereas diIPSCs should not be present in these PCs if FFI is laterally wired from individual GCs. However, we found single GC‐elicited diIPSCs with similar probabilities irrespective of the presence of monosynaptic excitation. This observation suggests that the wiring of FFI between individual GCs and PCs is independent of the direct excitation. Therefore, the randomly distributed FFI contributes to the hippocampal signal sparsification by setting the general excitability of the CA3 depending on the overall activity of GCs.

## 1 INTRODUCTION

**Figure 1:**
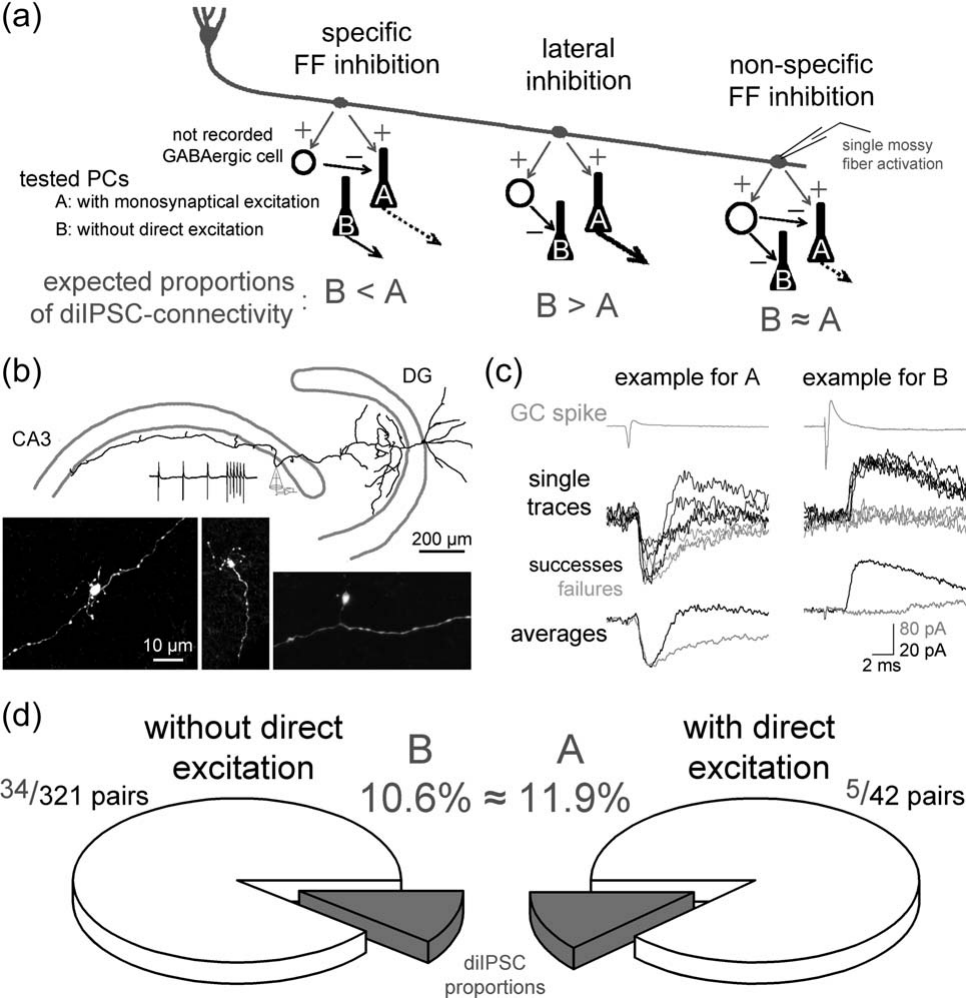
Wiring of FFI between individual cells of the DG‐CA3 interface. (a) Schematic figure representing the three alternative hypotheses for the possible wiring arrangements of the DG‐CA3 FFI involving individual GCs, PCs, and intermediate FF‐INs with excitatory and inhibitory synaptic connections. The letters A and B depict the two PC groups, which received or lacked monosynaptic EPSCs from the recorded MF track, respectively. The expected relationships between the relative proportions of pairs with diIPSC connections in the three wiring schemes are shown below (e.g., B < A, B > A, B ≈ A). Thicker or, dashed arrows, or the absence of arrows originating from the PCs indicate stronger, temporally structured, or inhibited CA3 PC output, respectively. (**b)** Drawing of the partially recovered presynaptic axons and dendrites of a DG GC. One of its large terminals was recorded in cell‐attached configuration and was subsequently in whole‐bouton mode to load with biocytin for the anatomical recovery. The presynaptic spikes at two frequencies during cell attached simulation are shown on top. (**c)** Example traces of MF terminal and PC pairs with diIPSC connectivity only (i.e., positive example for PCs in group B) and with both monosynaptic EPSC and diIPSC connectivity (i.e., group A). The upper gray traces show the average presynaptic action currents in the giant bouton in cell‐attached mode, the middle traces are individual postsynaptic responses or failures, and the trace pairs at the bottom show the averages of all traces with (black) or without (gray) diIPSCs. The morphology of one of the presynaptic GCs (right example) is shown on panel B. For further details about properties of the diIPSCs see **Supporting Information Fig. S1** and **Table S1**. The example traces were recorded with 20 Hz stimulation (for further analyses about the frequency‐dependence of the diIPSCs see **Supporting Information Fig. S2**). (**d)** Pie charts summarizing the numbers and probabilities of detecting diIPSCs between single MFs or GCs and PC pairs. The tested PCs either received monosynaptic EPSCs from the stimulated MF (group A, right pie) or lacked direct excitatory connections (group B, left pie). This arrangement led to similar probabilities of finding diIPSCs in the two groups (*p* = .791, Fisher's exact test). For further analysis using a bootstrapping method see **Supporting Information Fig. S3**

Feedforward inhibition (FFI) is a fundamental wiring scheme present in several cortical areas and is necessary for accurate neuronal information transfer (Buzsaki, 1984; Lawrence and McBain, 2003; Pouille and Scanziani, 2001). The dentate gyrus (DG)‐CA3 interface within the hippocampus is a prototypical FFI circuit due to its anatomical and physiological specializations (Acsády, Kamondi, Sik, Freund, & Buzsaki, 1998; Rollenhagen et al., 2007). Granule cells (GCs) innervate more GABAergic cells than pyramidal cells (PCs), and their strong excitation by sporadic GC firing suggests that local CA3 GABAergic inhibitory cells have prominent roles in limiting and shaping the excitation imposed by GCs onto CA3 PCs (Acsády et al., 1998; Henze, Wittner, & Buzsaki, 2002; Lawrence, Grinspan, & McBain, 2004; Szabadics and Soltesz, 2009; Toth, Suares, Lawrence, Philips‐Tansey, & McBain, 2000). Taking advantage of single MF‐triggered disynaptic inhibitory events (see below), as a hallmark of FFI we focused on only one aspect of the FFI: Does single GC‐triggered FFI prefer or avoid the PCs that the same GC directly excites? It is essential to know the manner in which FFI is wired at the level of individual cells, as different single‐cell connectivity arrangements can underlie different FFI functions (Acsády and Kali, 2007). Specifically, three connectivity scenarios are hypothesized that allow fundamentally different theoretical contributions (Figure 1a): (1) FFI and direct excitation specifically converge on individual PCs to form ensemble‐specific FFI, which would enable the precise temporal modulation of specific information streams from the DG to the CA3 (Pouille and Scanziani, 2001). In this arrangement, an individual feedforward interneuron (FF‐IN) is innervated by a presynaptic mossy fiber (MF), which specifically excites those PCs that receive concurrent inhibition from this FF‐IN. (2) Lateral inhibitory wiring would lead to potent and uncompromised excitation of a small subset of PCs over the majority of inhibited neighbors because, in this scheme, individual FF‐INs purposely avoid inhibiting PCs that share the same MF innervation as excitation source. (3) FF‐INs may nonselectively choose their PC targets (i.e., independent of excitatory afferent connectivity) and dynamically set the level of excitability of a large population of PC (Ferrante, Migliore, & Ascoli, 2009; Pouille and Scanziani, 2001). This arrangement provides optimal signal‐to‐noise communication between the DG and the CA3 at the lowest wiring and developmental cost while maintaining diverse plasticity opportunities (Buzsaki, Geisler, Henze, & Wang, 2004; Lawrence and McBain, 2003). Despite these functionally diverging contributions, we currently have no information regarding the predominant elementary FFI connectivity rule at the DG‐CA3 interface.

## 2 RESULTS AND DISCUSSION

To distinguish between the above possibilities and to gain insight into the preferred tripartite connectivity between individual MFs, FF‐INs, and PCs, we recorded disynaptic inhibitory postsynaptic currents (diIPSCs) in PCs evoked by a single MF, which was activated by either directly recording from a visually identified MF terminal (Figure 1b; Szabadics and Soltesz, 2009) or a GC in the CA3 (CA3 GC) (Szabadics, Varga, Brunner, Chen, & Soltesz, 2010). These disynaptic events reflect the reliable activation of an intermediate, not recorded FF‐IN by a single GC input. This locally activated FF‐IN, in turn, provides characteristic inhibitory events to the recorded PC (Figure 1c**, Supporting Information** Fig. S1) within a time window that is consistent only with two synaptic steps (2–7 ms; Brown and Johnston, 1983; Mori, Abegg, Gahwiler, & Gerber, 2004; Szabadics et al., 2010). Convergence of monosynaptic excitatory and disynaptic inhibitory connections in a single PC indicates that the individually recorded GC innervates both this PC and a FF‐IN and the latter also innervates the same PC. To detect both the depressing and the less reliable, facilitating disynaptic activations (Lawrence et al., 2004; Szabadics and Soltesz, 2009; Torborg, Nakashiba, Tonegawa, & McBain, 2010; Toth et al., 2000), the presynaptic stimulation protocol consisted of three low‐frequency action potentials (APs) at 20 Hz, followed by a sustained high‐frequency train (6 or 15 APs at 150 Hz). Thus, diIPSCs are sufficient tools to directly assess the single GC‐initiated FFI. To address the three hypotheses presented above, the recorded PCs were assigned to one of two groups based on the presence (*n* = 42 tested pairs) or absence (*n* = 321 tested pairs) of monosynaptic excitatory postsynaptic currents (EPSCs) from the simultaneously recorded MF or CA3 GCs. In these experimental arrangements, the testable assumptions are that (1) if the FFI is ensemble‐specific, then the proportion of diIPSC connectivity is higher in the monosynaptically excited PC group; (2) in case of lateral inhibition, a lack of diIPSC connections is expected in the directly innervated PCs; and (3) similar diIPSC proportions and similar properties are expected if the FFI is randomly distributed from individual GCs.

We found that the probabilities of observing single‐MF‐elicited diIPSCs were similar in the two groups of PCs (5 diIPSC couplings out of 42 tested pairs with monosynaptic EPSCs, 11.9%; 34 diIPSC couplings out of 321 tested pairs without monosynaptic EPSCs, 10.6%; Figure 1d). Statistical comparisons using the binomial test‐based Fisher's exact test did not reject the hypothesis that the proportions of diIPSC connections in the two groups were similar (*p* = .791, the estimated difference between the sample *p* values was –.013; the 95% confidence interval was between 0.0904 and −0.1167). The prevalence of diIPSCs was similar when we considered only those pairs wherein the presynaptic recording was made on giant MF terminals, which presumably originate from DG GCs (3 pairs with dual, disynaptic IPSC and monosynaptic EPSC connections out of 32 tested pairs with monosynaptic EPSCs, 9.4%; and 10 diIPSC connections out of 112 tested pairs without monosynaptic EPSCs, 8.9%). Thus, the directly innervated PCs are neither spared nor preferred by single MF‐evoked FFI. This is inconsistent with hypotheses #1 and #2 and supports the hypothesis #3, which states that the FFI is randomly distributed between the DG and the CA3.

For additional analysis on the accuracy of the obtained data size using bootstrap resampling, see **Supporting Information** Fig. S3. It is important to note that the relative numbers of the directly excited PCs in our sample are not representative. This is because we intentionally targeted likely connected MF‐PC pairs to increase the number of observations for both PC groups. Nevertheless, this aspect of our approach does not affect the unbiased sampling of FFI and diIPSCs. In triplet, quadruplet and quintuplet‐recordings, wherein 2, 3, or 4 PCs were tested with the same presynaptic MF source and at least one of them received a diIPSC, the prevalence of the diIPSCs in the other concurrently tested PCs (considering 12 pairs) were apparently higher (5 out of 12) than in the complete pool of data, as expected from the highly divergent innervation of PCs by feedforward inhibitory cells (Acsády et al., 1998; Bezaire and Soltesz, 2013). Furthermore, the properties of the diIPSC events were similar regardless of the presence of monosynaptic EPSCs from the same single GC source (**Supporting Information** Fig. S2 and Table S1). Thus, not only the wiring probability, but also the strength and kinetics of the inhibition (thus, the source of FFI; see discussion) appear to be similar in excited and not excited PCs. The properties of the monosynaptic EPSCs were also similar between pairs with both inputs and pairs with EPSCs only (**Supporting Information** Table 2). Thus, the PCs that are readily and strongly inhibited following GC activity seem to be similarly excited to those wherein no diIPSCs were detected.

Our findings reveal that individual MFs recruit CA3 PCs and FF‐INs regardless of the presence of inhibitory synaptic connections between them. Therefore, FFI between the DG and the CA3 is not wired to specifically inhibit a restricted population of PCs selected based on direct excitation from the GCs. This is consistent with the idea that the FFI is randomly distributed by individual GCs, which allows for the adjustment of general excitability of the CA3 network based on the activity of the DG. Our results from a sufficiently large sample size (**Supporting Information** Fig. S3) confirm the previous observations in slice cultures indicating disynaptic inhibition both in directly connected PCs and in PCs that are not excited by the same GC (Mori, Gahwiler, & Gerber, 2007).

Importantly, FFI is effective in preventing spike transmission during sparse GC firing, while high‐frequency GC bursts remain effective (Acsády and Kali, 2007; Henze et al., 2002; Zucca et al., 2017). This is because the large number of synaptic release sites (Rollenhagen et al., 2007) and activity‐dependent facilitation of release (Salin, Scanziani, Malenka, & Nicoll, 1996; Toth et al., 2000) provide an almost unlimited source of excitation from each giant MF bouton onto the PCs. However, this is restricted to only a few PCs because each GC typically innervates 10–20 PCs (Acsády et al., 1998). Thus, the sparse excitation of the PCs is accompanied by highly divergent and random FFI from the same GC input source. Specifically, the targets of the FFI originating from single GCs are multiplied via two divergent steps. First, GCs innervate 4–5 times more INs than PCs (Acsády et al., 1998). These FF‐INs then usually innervate hundreds of PCs (Bezaire and Soltesz, 2013). The dynamically adjusted global threshold for PC recruitment by the local, MF‐driven GABAergic inhibition, thus, potentially contributes to the disambiguation of small differences in the physiological DG activity, such as single AP‐ or burst‐firing. Nevertheless, certain small ensembles consisting of PCs inhibited to a smaller degree may become relatively more sensitive to forthcoming presynaptic GC activity than the majority of the population. The randomness of the FFI wiring implies not only that the FFI sets the activation threshold for the majority of the PCs, but also that it contributes, to some extent, to the precise tuning of spike timing in dually innervated PCs (Pouille and Scanziani, 2001).

Some of our observations suggest that the various types of FF‐INs follow the random wiring rules in a similar manner. Most importantly, the kinetic properties of the diIPSCs are similar in both groups of PCs. Given the anatomical and synaptic diversities of the FF‐INs, which result in cell type‐specific kinetics for their IPSCs, if some FF‐IN types were preferentially recruited for specific FFI or for lateral inhibition (i.e., if they are more or less likely to be recruited onto the directly MF‐innervated PCs), the properties of the diIPSC responses were expected to differ in PCs with and without direct MF input. In contrast to these assumptions, we found no difference in the diIPSCs between the two groups of PCs. Further support for the homogeneous FF‐IN type contribution is provided by the observation that the occurrences of the diIPSC events were similar during the low‐ and high‐frequency stimulus trains in the two PC groups. This observation supports the similar recruitment of the various FF‐IN types onto both PC groups because different IPSC occurrences were expected to reflect the cell type‐specific short‐term plasticity of the excitation of the FF‐INs (Mori et al., 2004; Szabadics and Soltesz, 2009; Torborg et al., 2010; Toth et al., 2000).

The detected diIPSCs represent only a fraction of the available actual FFI. The observed diIPSCs are those that involve the most strongly excited and single MF‐activated FF‐INs under the less excitable slice conditions (Barth et al., 2016). Thus, the obtained diIPSC connectivity underestimates the total number of inhibited PCs by the single GC‐driven FFI under in vivo conditions. Thus, most likely, only a small population of PCs is devoid of FFI from single GCs, as there are relatively large numbers of GABAergic cells that are innervated by single MFs and large numbers of PCs that are innervated by all types of FF‐INs (Acsády et al., 1998). Importantly, in spite of the underestimation of the overall FFI in our experimental arrangement, observation of diIPSCs in PCs both with and without direct excitation is consistent with random FFI innervation rules. First, merely the presence of the diIPSCs shows that none of the two PC groups is completely avoided by FFI. Second, because the recorded GC/MF‐PC pairs were randomly chosen, the preservation of the connection onto and from the intermediate interneurons and the reliable activation of some of them cannot be preferentially influenced by the preparation conditions. Thus, it is reasonable to conclude that the FFI of PCs was independent of their direct excitation from the individually tested GC source within the intact circuit.

The random local wiring of FFI is crucial to understanding the functional contexts of the plasticity mechanisms that regulate the DG/CA3 interface, including the experience‐dependent structural plasticity (De Paola, Arber, & Caroni, 2003; Ruediger et al., 2011; Tashiro, Dunaevsky, Blazeski, Mason, & Yuste, 2003), short‐term dynamics (Szabadics and Soltesz, 2009; Torborg et al., 2010; Toth et al., 2000) and activity‐dependent balance (Mori et al., 2004) of the recruitment of inhibition (Acsády and Kali, 2007). Notably, the numbers of innervated FF‐INs, particularly parvalbumin‐expressing cells, are markedly altered in response to environmental enrichment and experience (Ruediger et al., 2011). Thus, considering our findings that FFI does not have to be maintained in a selected population of PCs, the learning‐induced structural plasticity of the FFI circuit might not require energy‐costly mechanisms to maintain its proper functions (this would have been necessary if each parvalbumin‐positive cell selected certain structurally or functionally defined groups of PCs). However, the randomness excludes the possibility that the FFI and its structural plasticity are required for the suppression or enhancement of specific information channels. Rather, the FFI sets the overall excitability of the network depending on the state, demands, and previous activity of the DG and CA3 networks. Future studies should also address whether the random wiring rule is preserved or compromised during disease states, such as epilepsy. If this rule persists in spite of insults, therapies that enhance FFI between the DG and CA3 circuits may not be useful tools for combating abnormal GC activity. These strong activities are unlikely to be restrained by the FFI. Similarly strong, but normal GC activities, such as bursts, constitute physiological DG functions (Diamantaki, Frey, Berens, Preston‐Ferrer, & Burgalossi, 2016; Pernia‐Andrade and Jonas, 2014), and they are able overcome the randomly distributed FFI, as evidenced by the ability of single GCs to drive postsynaptic PC firing (Henze et al., 2002). Thus, abnormal strong activities can also overcome the FFI if the capacity for facilitating excitation is maintained.

## 3 DETAILED METHODS

For acute hippocampal slice preparations, adolescent Wistar rats (postnatal day 21–35, both sexes) were deeply anesthetized using isoflurane (in accordance with the ethical guidelines of the Institute of Experimental Medicine Protection of Research Subjects Committee, 22.1/1760/003/2009), their brains were dissected, and 350‐μm‐thick slices were cut in ice‐cold artificial cerebrospinal fluid (ACSF) containing (in mM): 85 NaCl, 75 sucrose, 2.5 KCl, 25 glucose, 1.25 NaH_2_PO_4_, 4 MgCl_2_, 0.5 CaCl_2_, and 24 NaHCO_3_. The slices were cut perpendicular to the axis of the hippocampus at its medial part and were parallel to the MFs to preserve the connections targeting cells in the CA3. The slices were incubated at 32 °C for 60 min after sectioning and were then stored at room temperature until they were used for recordings within 10 hr. For the recordings, the cells and the MF boutons were visualized using an upright microscope (Eclipse FN‐1, 40× 0.8NA water‐immersion objective; Nikon) with infrared (900 nm) Nomarksi differential interference contrast optics. The ACSF used for the recording was composed of (in mM): 126 NaCl, 2.5 KCl, 26 NaHCO_3_, 2 CaCl_2_, 2 MgCl_2_, 1.25 NaH_2_PO_4_, and 10 glucose. All recordings were carried out at 34–36 °C using MultiClamp 700B amplifiers (Molecular Devices).

Postsynaptic PCs were voltage clamped at −55 to −45 mV, which is above the Cl^–^ reversal potential, for the reliable distinction of EPSCs from IPSCs and to avoid reaching the AP threshold. We used a low [Cl^–^] intracellular solution, which was composed of (in mM): 133.5 K‐gluconate, 1.8 NaCl, 1.7 MgCl_2_, 0.05 EGTA, 10 HEPES, 2 Mg‐ATP, 0.4 Na_2_‐GTP, 10 phosphocreatine‐disodium (pH 7.25). Note that the tested PCs were not biocytin labeled. When the monosynaptic EPSC amplitudes were very large (potentially masking diIPSC events), the PCs were depolarized further toward the reversal potential of the EPSCs to obtain sufficient resolution for the smallest outward IPSCs. Only those recordings wherein spontaneous outward IPSCs appeared were considered for analysis. Series resistance (5–30 MΩ) was monitored by the capacitive artifact in response to a 5‐mV step in each trace. PCs were identified based on their characteristic firing pattern and membrane properties (Szabadics et al., 2010).

Presynaptic giant MF terminals were preferentially assessed in cell‐attached configuration (*n* = 126 pairs) with pipettes containing intracellular solution (Szabadics and Soltesz, 2009) to better preserve their synaptic release (Vyleta and Jonas, 2014). MF boutons were targeted on or near the apical dendrites of the recorded PCs to facilitate the recording of monosynaptic pairs. To provide unbiased recording criteria, presynaptic MF boutons and postsynaptic PCs were recorded at 30–50 µm from the surface of the slice. Presynaptic CA3 GCs were 30–100 µm deep to avoid cut axons. In some cases, presynaptic MFs were tested with multiple PCs (*n* = 82 MFs with 2 PCs, *n* = 12 with 3 PCs, *n* = 6 MFs with 4 PCs).

After testing the connections from the cell‐attached recorded MF terminals (at least 5 traces were tested) and recording the evoked responses (if detected), we attempted to break the membrane to establish the whole‐cell recording configuration (either voltage‐ or current‐clamped) in order to obtain biocytin labeling of the recorded MF and its parent soma. Some giant MF terminals were recorded in current clamp mode from the beginning without cell‐attached mode recordings (*n* = 18). Presynaptic CA3 GCs were always recorded using somatic current clamp. The intracellular solution for presynaptic axonal and somatic recordings contained (in mM): 90 K‐gluconate, 43.5 KCl, 1.8 NaCl, 1.7 MgCl_2_, 0.05 EGTA, 10 HEPES, 2 Mg‐ATP, 0.4 Na_2_‐GTP, 10 phosphocreatine‐disodium, and 8 biocytin (pH 7.25).

The diIPSCs were analyzed within a predefined time window (2–7 ms from the peak of the presynaptic AP), when all events were counted. The analysis potentially included a few spontaneous events originating from other presynaptic sources. The simultaneously recorded MFs, CA3 GCs and PCs were within 200 µm in both groups. A diIPSC connection was accepted only if the evoked events were clearly more frequent than expected from spontaneous rates and had consistent properties (amplitude, delay, and kinetics). All traces were analyzed by at least two investigators. Note the slightly higher probability of detecting diIPSCs from presynaptic CA3 GCs compared to DG MFs (11.8%, 26 out of 219 CA3GC pairs vs. 9.03%, 13 out of 144 MF pairs). This may have been due to the longer “allowed” recording duration wherein active FF‐INs were more likely to be detected. Regardless of the above observation, the probabilities of the diIPSCs were similar when presynaptic CA3 GCs with or without connections to PCs and presynaptic MF without or without connection to PCs were tested. Furthermore, the delays, decay time constants, potencies and probabilities of CA3 GC‐ and MF‐triggered diIPSCs were similar (**Supporting Information** Fig. S1). The delays were measured from the peak of the presynaptic APs (whole cell recordings) or from the negative peak of the action currents (in bouton attached recordings) to the onset of events. Note that a slight bias is likely to be introduced by the different presynaptic conditions. However, none of the conclusions are affected by this error in the latency measurements because the AP rising phase is almost two orders of magnitudes faster than the delays of the diIPSCs. The 10–90% rise times were measured for individual events, while the decay was fitted to averages using a single exponential.

After the recordings, the slices were fixed for one day in a 0.1 M phosphate buffer solution containing 2% paraformaldehyde and 0.1% picric acid at 4 °C. The slices were resectioned into 60 μm‐thick sections and treated with 0.3% Triton X‐100 and 10% normal goat serum. The biocytin labeling, which was loaded into the presynaptic axons or cells was revealed using Alexa Fluor 488‐conjugated streptavidin (1:500) by epifluorescence or confocal microscopy.

## ACKNOWLEDGMENTS

This work was funded by the Wellcome Trust (International Senior Research Fellowship #087497), the Hungarian Academy of Sciences (Lendület Initiative #LP‐2009–009), the Hungarian Brain Research Program (KTIA_13_NAP‐A‐I/5) and the Stephen W. Kuffler Research Scholarship (to V.J.O.). The authors thank José Guzmán, Attila Gulyás and Mark Eyre for their valuable comments on the manuscript. They thank Dóra Kókay for technical assistance. They would like to thank the kindly provided microscopy support by László Barna, Csaba Pongor and Judit Veres at the Nikon Microscopy Center at the Institute of Experimental Medicine, which is sponsored by Nikon Europe, Nikon Austria and Auro‐Science Consulting. N.M., V.J.O., and J.B. were students at the János Szentágothai Doctoral School of Neurosciences, Semmelweis University.

## CONFLICT OF INTEREST

The authors have no conflict of interest to declare.

## Supporting information

Supporting Information
