# Supplementary material for: Feedforward inhibition is randomly wired from individual granule cells onto CA3 pyramidal cells
Source: Hippocampus. 2017 Jul 24;27(10):1034–9. doi: 10.1002/hipo.22763 (PMC5637936; doi:10.1002/hipo.22763)
Supplement: Supplementary file 1 — Supporting Information [file HIPO-27-1034-s001.docx]

# Supporting Information Figure 1





**Comparison of the properties of the diIPSCs evoked by the two different MF sources.**

**A**. Comparison of the probabilities of diIPSCs evoked by CA3 GCs (light gray columns) or MFB recordings (gray columns) stimulated with 3 APs at 20Hz or 6 APs at 150Hz (ANOVA, frequency dependency: p = 0.00408, “presynaptic” sources: p = 0.63927, n = 12 and 22 MF-PC and CA3 GC-PC pairs, all tested with both frequencies). **B**. Comparison of the delays and kinetics of the diIPSCs evoked by MFB and CA3 GCs (Mann-Whitney test, p = 0.463, p = 0.025, and p = 0.441, for delay, rise time and decay, respectively, n = CA3 GC pairs and n = MFB pairs).

# Supporting Information Figure 2

**

**

**Frequency-dependent properties of the diIPSCs.**

**A**. Frequency dependence of the diIPSCs during 20Hz 3 AP-trains and 150Hz 15 AP-trains (n = 21 pairs). As expected from the known short-term plasticity of the MF-to-FF-IN EPSCs the highest probability of diIPSCs were at the onset of the 150Hz trains. **B.** Properties of the individual diIPSCs. Each symbol indicates individual experiments. Open symbols are diIPSC-only connections and filled symbols show the properties of diIPSCs in dual diIPSC+monoEPSCs connections.

# Supporting Information Figure 3

**
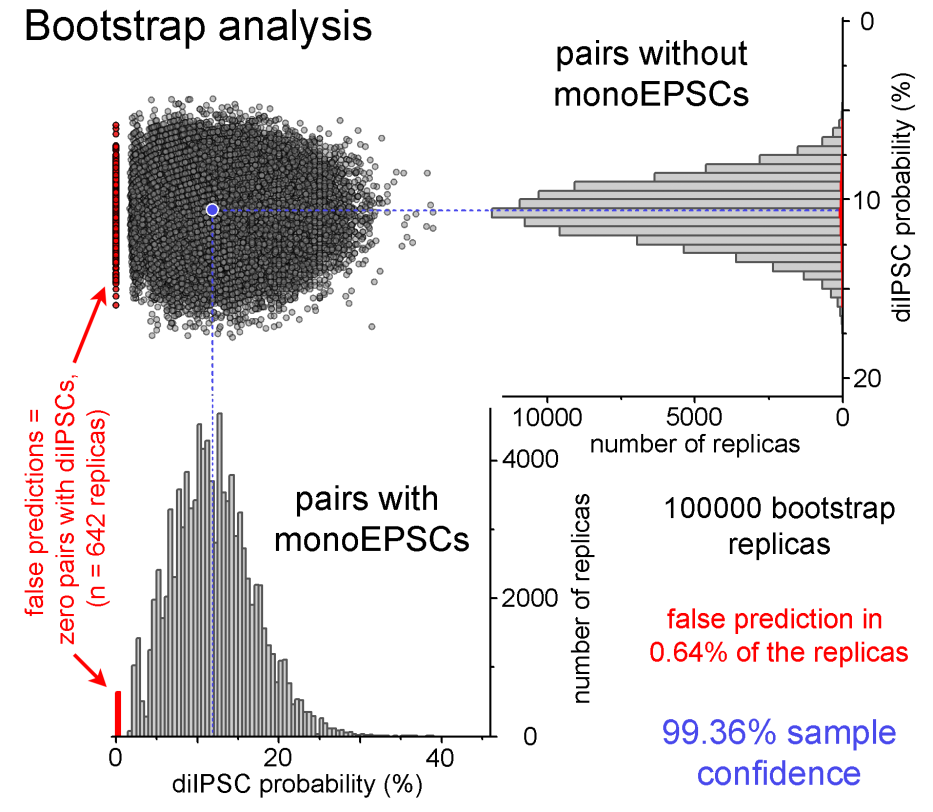
**

**Bootstrap resampling of the experimental data set.**

In order to test the accuracy of the predictions of the obtained data, we simulated the incidences of diIPSC-connectivity among the directly excited (A group in Figure 1A) and not excited PCs (B group) using bootstrapping approach in 100000 replicas. These replicas were the resampling from the experimental pair data (n = 42 pairs with direct excitation out of which 5 was also connected by diIPSCs, and n = 321 pairs without direct excitation with 34 diIPSCs). Specifically, we asked the question how likely is that the observed experimental data falsely predict random connectivity, while the underlying biological connectivity was either specific or lateral inhibition. We considered replicas false prediction (red symbols) if the resampled data did not contain any diIPSC-connectivity between monosynaptically connected MF-PC pairs (never occurred) or between not connected MF-PC pairs (642 replicated cases). Transparent gray symbols indicate the replicas in which diIPSCs occurred in both groups of tested PCs (99358 replicated cases). Blue circle indicates the experimentally observed ratios. Notice the different diIPSC probability axis for the two data sets. Thus, a sample size as our recorded data sets predicts the connectivity with 99.36% confidence.

# Supporting Information Table 1.

| Mean ± s.e. (Median) | diIPSC only connections | diIPSC in dual connections | MW-test p value |
| --- | --- | --- | --- |
| **Delay from AP (ms)** | 3.53 ± 0.12 (3.53) | 4.11 ± 0.31 (4.33) | 0.104 |
| **Variance of delay (ms^2^)** | 1.18 ± 0.14 (1.18) | 1.04 ± 0.38 (0.8) | 0.808 |
| **Rise time (ms)** | 0.93 ± 0.05 (0.85) | 0.76 ± 0.11 (0.81) | 0.398 |
| **Decay time constant (ms)** | 8.40 ± 0.43 (8.03) | 8.88 ± 0.55 (8.88) | 0.450 |
| **Potency (pA)** | 34.8 ± 2.8 (33.6) | 70.5 ± 37.3 (37.9) | 0.375 |

Comparison of the properties of the diIPSCs (n = 30 diIPSC only connections and n = 4 diIPSC in dual connections).

# Supporting Information Table 2.

| Mean ± s.e. (Median) | EPSC only connections | EPSC in dual connections | MW-test p value |
| --- | --- | --- | --- |
| **Delay from AP (ms)** | 0.61 ± 0.03 (0.57) | 0.71 ± 0.08 (0.63) | 0.224 |
| **Rise time (ms)** | 0.89 ± 0.04 (0.82) | 1.02 ± 0.11 (0.95) | 0.178 |
| **Decay time constant (ms)** | 6.41 ± 0.37 (5.95) | 7.48 ± 0.89 (6.83) | 0.213 |
| **Facilitation (3^rd^/1^st^ 20Hz-AP)** | 1.33 ± 0.10 (1.26) | 1.44 ± 0.31 (1.23) | 0.903 |
| **Facilitation (150Hz/20Hz charge)** | 6.92 ± 1.30 (5.02) | 6.79 ± 0.08 (6.79) | 0.405 |

Comparison of the properties of the EPSCs in pairs with or without diIPSCs (n = 28 EPSC only connections and n = 4 EPSC in dual connections).
